# Soluble aggregates present in cerebrospinal fluid change in size and mechanism of toxicity during Alzheimer’s disease progression

**DOI:** 10.1186/s40478-019-0777-4

**Published:** 2019-07-26

**Authors:** Suman De, Daniel R. Whiten, Francesco S. Ruggeri, Craig Hughes, Margarida Rodrigues, Dimitrios I. Sideris, Christopher G. Taylor, Francesco A. Aprile, Serge Muyldermans, Tuomas P. J. Knowles, Michele Vendruscolo, Clare Bryant, Kaj Blennow, Ingmar Skoog, Silke Kern, Henrik Zetterberg, David Klenerman

**Affiliations:** 1 Department of Chemistry, University of Cambridge, Cambridge, CB2 1EW UK; 2 Centre for Misfolding Diseases, University of Cambridge, Cambridge, CB2 1EW UK; 3 Department of Veterinary Medicine, University of Cambridge, Cambridge, CB3 0ES UK; 4 Laboratory of Cellular and Molecular Immunology, Vrije Universiteit Brussel, Brussels, Belgium; 5 Clinical Neurochemistry Laboratory, Unit of Department of Psychiatry and Neurochemistry, Institute of Neuroscience and Physiology, the Sahlgrenska Academy at the University of Gothenburg, Göteborg, Sweden; 6 Clinical Neurochemistry Laboratory, Sahlgrenska University Hospital, Mölndal, Sweden; 7 Neuropsychiatric Epidemiology Unit, Department of Psychiatry and Neurochemistry, Institute of Neuroscience and Physiology, the Sahlgrenska Academy at the University of Gothenburg, Göteborg, Sweden; 8 Department of Neurodegenerative Disease, UCL Queen Square Institute of Neurology, University College London, Queen Square, London, UK; 9 UK Dementia Research Institute at University College London, London, UK; 10 UK Dementia Research Institute at University of Cambridge, Cambridge, CB2 0XY UK

**Keywords:** Alzheimer’s disease, Mild cognitive impairment, Cerebrospinal fluid, Protein aggregation, Structure-function relation, Super-resolution imaging, Disease mechanism

## Abstract

Soluble aggregates of amyloid-β (Aβ) have been associated with neuronal and synaptic loss in Alzheimer’s disease (AD). However, despite significant recent progress, the mechanisms by which these aggregated species contribute to disease progression are not fully determined. As the analysis of human cerebrospinal fluid (CSF) provides an accessible window into the molecular changes associated with the disease progression, we characterised soluble aggregates present in CSF samples from individuals with AD, mild cognitive impairment (MCI) and healthy controls using a range of sensitive biophysical methods. We used super-resolution imaging and atomic force microscopy to characterise the size and structure of the aggregates present in CSF and correlate this with their ability to permeabilise lipid membranes and induce an inflammatory response. We found that these aggregates are extremely heterogeneous and exist in a range of sizes, varying both structurally and in their mechanisms of toxicity during the disease progression. A higher proportion of small aggregates of Aβ that can cause membrane permeabilization are found in MCI CSF; in established AD, a higher proportion of the aggregates were larger and more prone to elicit a pro-inflammatory response in glial cells, while there was no detectable change in aggregate concentration. These results show that large aggregates, some longer than 100 nm, are present in the CSF of AD patients and suggest that different neurotoxic mechanisms are prevalent at different stages of AD.

**Electronic supplementary material:**

The online version of this article (10.1186/s40478-019-0777-4) contains supplementary material, which is available to authorized users.

## Introduction

Small soluble aggregates of amyloid-β (Aβ) have been shown to impair hippocampal synaptic plasticity, induce learning deficits and correlate with cognitive impairments both in Alzheimer’s disease (AD) mouse models and humans [13, 27, 30, 40]. These soluble aggregates can exert cellular toxicity via a range of diverse mechanisms, including oxidative stress, disruption of Ca^2+^ homeostasis and cellular signalling, mitochondrial alterations, glial activation and inflammation [2, 18]. Many of these processes are the consequence of two fundamental upstream events induced by soluble aggregates: *(i)* permeabilisation of cell membranes by non-specific binding [6, 15] and *(ii)* specific interactions with receptors in cell membranes [7, 18]. It is also known that the morphology of protein aggregates can determine the level of their involvement in different biological interactions. Hydrophobic protein aggregates more readily interact with hydrophobic lipid membranes, while the size and shape of the aggregates determine the affinity of binding to receptors [6, 7, 18, 25]. Understanding how the intrinsic heterogeneity in the size, shape and structure of the aggregates present in the human brain influences their mechanism of toxicity and how such heterogeneity changes as AD progresses is crucial in identifying the molecular pathways that lead to neuronal death.

Most of our current knowledge about the origins and morphologies of the toxic soluble species involved in neurodegenerative mechanisms is derived from in vitro studies and animal models. These studies have shown the presence of soluble aggregates with large heterogeneity in size (dimer to higher order multimers), shape (small spherical to fibril like) and structure (random coil to β-sheet) [2, 40]. During disease progression, Aβ in the human brain can aggregate into a large number of different forms, consisting of different numbers of peptides, sizes, shapes and structural configurations, with multiple possible post-translational modifications and co-aggregating species. One way to characterise the presence of toxic aggregates at different clinical stages and its implication on disease progression is to study these soluble aggregates present in AD, mild cognitive impairment (MCI) and healthy control cerebrospinal fluid (CSF), since this fluid can reflect at least some of the biochemical changes occurring inside the brain. MCI is a heterogeneous syndrome, which may have many underlying causes [39]. A proportion of MCI patients have AD pathology and are at increased risk of developing AD dementia. MCI can thus be conceptualised as a prodromal state in the AD continuum - a transition between normal cognitive aging and AD [5, 39]. In this study, we used the core AD CSF biomarkers and clinical dementia rating (CDR), which depend on the cognitive ability of individuals (see Methods), to distinguish the MCI cases that had Alzheimer’s pathologic changes. These biomarkers used are strongly predictive of the patient having MCI or AD and the agreement is 89–90% [17]. This is also in good agreement with Positron-emission tomography (PET) imaging classification of AD [17]. We have selected MCI cases based on the low level of Aβ42 (Aβ42 < 600 ng/L) indicating brain amyloidosis and where the CDR is 0.5. This procedure allowed us to select solely for the MCI cases with brain amyloidosis [21], which will henceforth be referred to as MCI. All the healthy controls are free of MCI and dementia and had a CDR of 0. Therefore, to understand the nature of soluble aggregates present at different stages of AD and how they induce cellular toxicity, we studied CSF samples collected from individuals affected from AD and MCI and compared these with healthy controls.

CSF is continuously produced, recycled and freely exchanged with the interstitial fluid in the brain, making it an ideal reservoir of soluble aggregates that can be reflective of toxic species present in the brain tissue for that disease stage. It has been demonstrated that soluble Aβ is secreted into the CSF, making it an ideal biomarker candidate for AD [4, 24, 39]. The total mass of Aβ oligomers and monomers have been measured in AD CSF using sensitive enzyme-linked immunosorbent assay (ELISA) based methods [4, 22, 38] . In all experiments there is significant overlap in the total mass of Aβ oligomers between control and AD patients, although a small increase in Aβ oligomers mass has been reported for some cohorts [22, 38]. This suggest that structural changes in the Aβ oligomers may be more important in driving disease. However, due to a lack of suitably sensitive methods, the specific detection and quantification of the heterogeneity in morphology of soluble Aβ aggregates present in CSF have not yet been determined. Understanding the changes in these soluble aggregates that occur during disease progression may provide new insights into the disease mechanisms with potential for early diagnosis.

## Materials and methods

### Ad CSF

The CSF used for all the assays was collected by lumbar puncture from patients who sought medical advice because of memory problems. The samples were de-identified and aliquoted into 0.5 mL aliquots in polypropylene cryo tubes following centrifugation at 2,200 x*g* in 20 °C for 10 min and stored at − 80 °C pending experimental use. CSF Aβ1–42, T-tau and P-tau181 were quantified with sandwich ELISAs INNOTEST® β-amyloid_1–42_, hTAU-Ag and Phospho-Tau [181P], respectively (Additional file 1: Table S1). All measurements were performed in clinical laboratory practice by board-certified laboratory technicians using procedures approved by the Swedish Board for Accreditation and Conformity Assessment. Intra-assay coefficients of variation were below 10%. All AD-positive samples had protein levels of Aβ42 < 600 ng/L, T-tau > 350 ng/L and P-tau181 > 80 ng/L. The study protocol was approved by the regional ethics committee at the University of Gothenburg.

### MCI and control CSF

MCI (6) and control (6) samples were collected from the Gothenburg H70 Birth Cohort Studies in Gothenburg, Sweden [24, 37]. These samples were obtained from the Swedish Population Registry and included both persons living in private households and in residential care and approved by the Regional Ethical Review Board in Gothenburg. As described previously, lumbar punctures to collect CSF samples were performed in the L3/L4 or L4/L5 inter-space in the morning [3]. The first 10 mL of CSF were collected in a polypropylene tube and immediately transported to the laboratory for centrifugation at 1,800 x*g* in 20 °C for 10 min and stored at − 80 °C pending experimental use.. The supernatant was gently mixed to avoid possible gradient effects, aliquoted in polypropylene tubes and stored at − 70 °C [3]. CSF Aβ42, T-tau and P-tau181 were quantified with sandwich ELISAs INNOTEST® β-amyloid_1–42_, hTAU-Ag and Phospho-Tau [181P], respectively.

As previously published, every 70-year-old in Gothenburg, Sweden, born during 1944 on prespecified birthdates was invited to the examination in 2014–2016, and 1203 participated (response rate 72.2%). Of these, 430 (35.8%) consented to a lumbar puncture [24]. Participants were examined at the Neuropsychiatric memory clinic at Sahlgrenska University Hospital in Gothenburg or at home. Experienced psychiatric research nurses performed the neuropsychiatric examinations, which comprised ratings of psychiatric symptoms and signs, tests of mental functioning, including assessments of episodic memory (short-term, long-term), aphasia, apraxia, agnosia, executive functioning and personality changes. Key informant interviews were performed by psychiatric research nurses as described previously [24, 37]. Examinations included the Mini Mental State Examination and the clinical dementia rating (CDR). Dementia was diagnosed according to the DSM-III-R criteria.

For each participant, the clinical MCI diagnosis was determined in a clinical consensus conference, comprised of a neurologist and psychiatrist, taking into account the participants history and information from informants. CSF biomarker levels of Aβ 42, total-tau and Phopho-tau were considered in choosing participants with MCI and underlying preclinical AD pathology. All MCI participants had a clinical dementia rating (CDR) score of 0.5. Healthy controls had to be free of MCI and Dementia and had a CDR of 0. Dementia diagnoses were an exclusion criterion in this study.

All samples were collected in the morning (before lunch) at the highly coordinated memory clinics in south-western Sweden, using clinically implemented CSF sampling protocol that has been detailed in: Blennow et al. [5]. All samples were stored in Gothenburg with only one freeze-thaw cycle.

### Membrane permeabilisation assay

A detailed protocol and further description of the membrane permeabilisation can be found elsewhere [14]. Briefly, 200 nm mean diameter vesicles composed of 16:0–18:1 PC and 18:1–12:0 biotin PC (100:1) (Avanti Lipids) were prepared using extrusion and freeze-thaw cycles. Vesicles were filled with 100 μM of Cal-520 dye and tethered in the glass surface via biotin-neutravidin linkage. A glass surface was coated using PLL-g-PEG and PLL-g-PEG biotin (10:1) (Susos AG). To measure the background for each set, 9 different images were acquired in the presence of only 30 μL Ca^2+^ containing buffer, which we denote as blank (F_blank_). Then a 30 μL CSF aliquot was added to the glass coverslip and incubated for 15 min before images of the exact same fields of view were recorded (F_sample_). Then, 10 μL of ionomycin was added to the same coverslip and images of the vesicles were acquired in the same fields of view (F_ionomycin_). The recorded images were analysed to determine the fluorescence intensity of each vesicle under the three different conditions and the average Ca^2+^ influx for each vesicle was calculated using the formula (F_sample_ - F_blank_) / (F_ionomycin_ - F_blank_) X 100%. The stage movements were performed using a bean shell-based program which allows to select fields of view without any user bias. For antibody experiment, CSF and 300 nM antibody were incubated for 15 min and added to the vesicle containing glass coverslip for membrane permeabilisation study.

Imaging of individual vesicles were performed using a home-built total internal reflection fluorescence (TIRF) microscope. For excitation, a 488 nm laser (Toptica) beam was focussed in the back focal plane of the 60x, 1.49 NA oil-immersion objective lens (APON60XO TIRF, Olympus, N2709400). Fluorescence emission from the dyes were collected by the same objective and imaged onto an air-cooled EmCCD camera (Photometrics Evolve, EVO-512-M-FW- 16-AC-110).

### Inflammation assay

The BV2 cell line was derived from immortalised murine neonatal microglia and grown in 10% foetal bovine serum and 1% L-Glutamine supplemented Dulbecco’s Modified Eagle’s (DMEM) medium. The cells were incubated at 37 °C in a humidified atmosphere of 5% CO2 and 95% air, until the cell density reached approximately 1.6 × 10^6^ cell/mL. 200 μL of CSF was diluted in 1 mL of DMEM and added to BV2 microglia cells. Every 24 h the supernatant was removed for analysis and replaced by fresh diluted CSF. The TNF-α concentration in the supernatant was quantified using the Duoset® enzyme-linked immunosorbent assay (ELISA) development system (R&D Systems, Abingdon, Oxfordshire, UK). Three wells for each CSF were used to estimate variation in the experiments. For antibody experiment, CSF and antibody were diluted in DMEM buffer and added to the vesicle containing glass coverslip.

### Aptamer-DNA PAINT (AD PAINT) imaging

AD PAINT was performed as described previously^22^. Briefly, glass-slides were cleaned with argon plasma, rinsed with 1% tween-20 and washed with PBS. All buffers were first passed through a 0.02 μm filter (Anotop25, Whatman). The CSF was diluted ten-fold in PBS and added to wells on the coverslip formed by a multiwell chamber coverslip (CultureWell CWCS-50R-1.0). After leaving any aggregates to adhere to the surface for 5 min the CSF was removed, the wells were washed with PBS and then filled with imaging mix (1 nM imaging strand (sequence CCAGATGTAT-CY3B) and 100 nM aptamer-docking strand (sequence GCCTGTGGTGTTGGGGCGGGTGCGTTATACATCTA) in PBS). The wells were then sealed using another clean coverslip. The samples were imaged on a home-built TIRF microscope using a 1.49 N.A., 60x TIRF objective (UPLSAPO, 60X, TIRF, Olympus) mounted on a Ti-E Eclipse microscope (Nikon) fitted with a perfect focus system. The Cy3B was excited at 561 nm (Cobalt Jive, Cobalt) passed through a FF01–561/14–25 excitation filter (Semrock). Fluorescence was separated from the excitation light using a dichroic mirror (Di01-R405/488/561/635, Semrock), passed through a filter (LP02-568RS-25, Semrock) and focussed on the EMCCD camera described above operating in frame transfer mode (electron-multiplying Gain of 11.5 e-1/ADU and 250 ADU/photon). To eliminate user bias an automated script (Micro-Manager [12]) was used to collect images in a grid. Five thousand frames were collected with an exposure time of 50 ms. Images were analysed using the Peak Fit ImageJ plugin of the GDSC Single Molecule Light Microscopy package and custom scripts written in Python. The data analysis is described in detail in Whiten et al [43]*.*

### Atomic force microscopy imaging

CSFs are diluted 10x using PBS buffer and image on freshly cleaved mica substrates using AFM. 10 μL diluted CSF samples were deposited on the substrate at room temperature. The samples were incubated for 10 min and followed by rinsing with 1 mL milli Q water. Then the samples were dried using a gentle flow of nitrogen gas. AFM maps were created using a NX10 (Park Systems, South Korea) and JPK nanowizard2 system (JPK Instruments, Germany) operating in non-contact mode. This set-up equipped with a silicon tip with a nominal radius of < 10 nm. Scanning Probe Image Processor (SPIP) (Image Metrology, Denmark) software were used for image flattening and single aggregate statistical analysis. The lateral resolution of this technique is determined by the geometry of the AFM tip, although the measurement of the height of individual species is not significantly affect by the tip geometry. The average level of noise for each image was measured using SPIP software and is well below 0.1 nm. The signal to noise ratio for measuring a single Aβ42 monomer is close to 10. Thus, the characterisation of cross-sectional height can be performed with high sensitivity and accuracy.

### Confocal imaging of CSF using pentameric formyl thiophene acetic acid (pFTAA)

Single-molecule confocal experiments were performed using the previously-reported method [41]. CSF samples were diluted 1:1 into pentameric formyl thiophene acetic acid (pFTAA) solution (60 μM, PBS) and withdrawn through a single-channel microfluidic device at a flow velocity of 0.56 cm·s^− 1^. A 488 nm laser beam (1.5~mW, Spectra Physics Cyan CDRH) was directed to the back aperture of an inverted microscope (Nikon Eclipse TE2000-U). The beam was reflected by a dichroic mirror (51008BS, Chrome) and focussed to a concentric diffraction-limited spot, 10 μm into the solutions in the microfluidic channel through a high numerical aperture oil immersion objective (Apochromat 60X, NA 1.40, Nikon). Fluorescence was collected using the same objective, passing through the same dichroic mirror and imaged onto a 50 μm pinhole (Melles Griot) to remove out-of-focus light. The emission was filtered (535AF45, Omega) and directed to an avalanche photodiode (APD, SPCM-14, Perkin-Elmer Optoelectronics). A custom-programmed field-programmable gate array, FPGA (Colexica), was used to count the signals from the APD and combine these into time-bins which were selected according to the expected residence time of molecules passing through the confocal probe volume. At each time-point data were collected for 10 min (100000-time bins, bin-width 0.2~ms). The experimental output data were collected using an FPGA card and analysed in Python using custom-written code. A threshold of background + 20 was set for all measurements so as to maximise the number of events, whilst removing the noise.

### Production and purification of antibodies

The Aβ-specific nanobody Nb3 was isolated from a llama (*Llama glama*) and amplified from the peripheral blood lymphocytes, as described previously [11]. The concentration was estimated by absorbance spectroscopy at 280 nM using a molecular extinction coefficient, which was calculated based on the sequence of the protein of 21,555 M^− 1^ cm^− 1^. Nb3 binds to the epitope 17–28 of Aβ with a measured K_d_ for the monomer of 13 nM [32] .

Rationally designed antibodies to Aβ42 were generated as previously described [1, 10]. These antibodies are designed to preferentially bind the target epitope when the protein is in the aggregated rather than in the monomeric conformation. Briefly, complementary peptides were selected using the cascade method to target linear epitopes within Aβ42 that scan its entire sequence. These complementary peptides were then grafted into the complementarity determining region 3 of an antibody scaffold by means of a mutagenic polymerase chain reaction with phosphorylated oligonucleotides. The DesAb variants were overexpressed and purified using pRSET-B vector in *E. coli* BL21 (DE3) Gold Strain. Overnight Express Instant TB Medium (Merck Millipore) supplemented with 100 μg/ml ampicillin were used as a medium for cell growing. Then it was harvested by centrifugation; resuspended in phosphate-buffered saline (PBS), then one tablet of EDTA-Free Complete Protease Inhibitor Cocktail (Roche) was added to 500 mL of cell growth media. Then cells are lysed by sonication and debris was removed by centrifugation at 15,000 rpm (JA-20 rotor, Beckman Coulter). The cleaned lysate was loaded onto a Ni^2+^ − NTA Superflow column (Qiagen) using PBS with 10 mM imidazole. Then His-tagged DesAbs were eluted with PBS containing 200 mM imidazole and lastly the. Imidazole was then removed using size exclusion chromatography. Protein concentration was determined by absorbance measurement at 280 nm using theoretical extinction coefficients and calculated with ExPASy ProtParam. Previous experiments have confirmed that the rationally designed antibodies bind to their target epitopes. The affinity for monomeric Aβ is in the range 200 to 900 nM, while the affinity for Aβ aggregates is in the range 1–10 nM [1, 10].

### Statistical tests

**Fig. 1:**
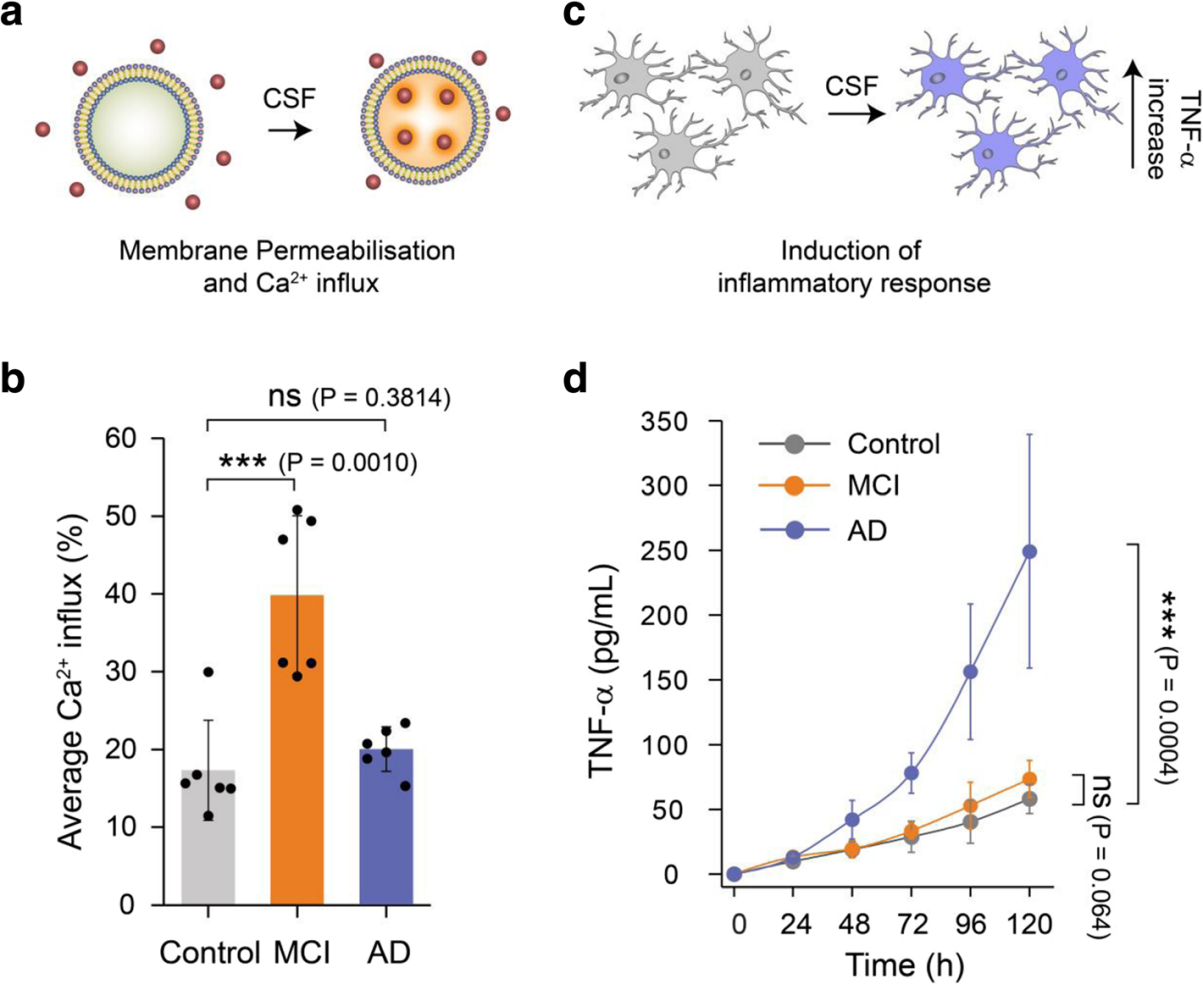
Soluble aggregates present in MCI and AD CSF samples show different dominant mechanisms of toxicity. **a** Membrane permeabilisation was measured by immobilising hundreds of vesicles containing a Ca^2+^-sensitive dye on PEGylated glass cover slides. If any species present in the CSF disrupts the integrity of the lipid membrane of the vesicles, Ca^2+^ ions from the surrounding buffer enter into individual vesicles in numbers that can be quantified using highly sensitive TIRF microscopy. **b** Aliquots of MCI CSF can cause more membrane permeabilisation compared to AD and control CSF (*n* = 6 AD, 6 MCI, 6 control CSF). A two-sample unpaired t-test was performed to compare each data set. **c** The inflammatory response in microglia cells was quantified using an ELISA assay to measure the levels of secreted tumour necrosis factor alpha (TNF-ɑ). **d** For this study, CSF samples were added to BV2 microglia cells and incubated for 120 h. Every 24 h the TNF-α concentration in the supernatant was quantified using an ELISA assay. AD CSF samples were more effective MCI and control CSF samples in inducing an inflammatory response (lines are guide to the eye; *n* = 10 AD, 6 MCI, 6 control CSF). Error bars are the standard deviation among data points. A two-sample unpaired t-test at the 120 h time point was performed to compare the data sets

**Fig. 2:**
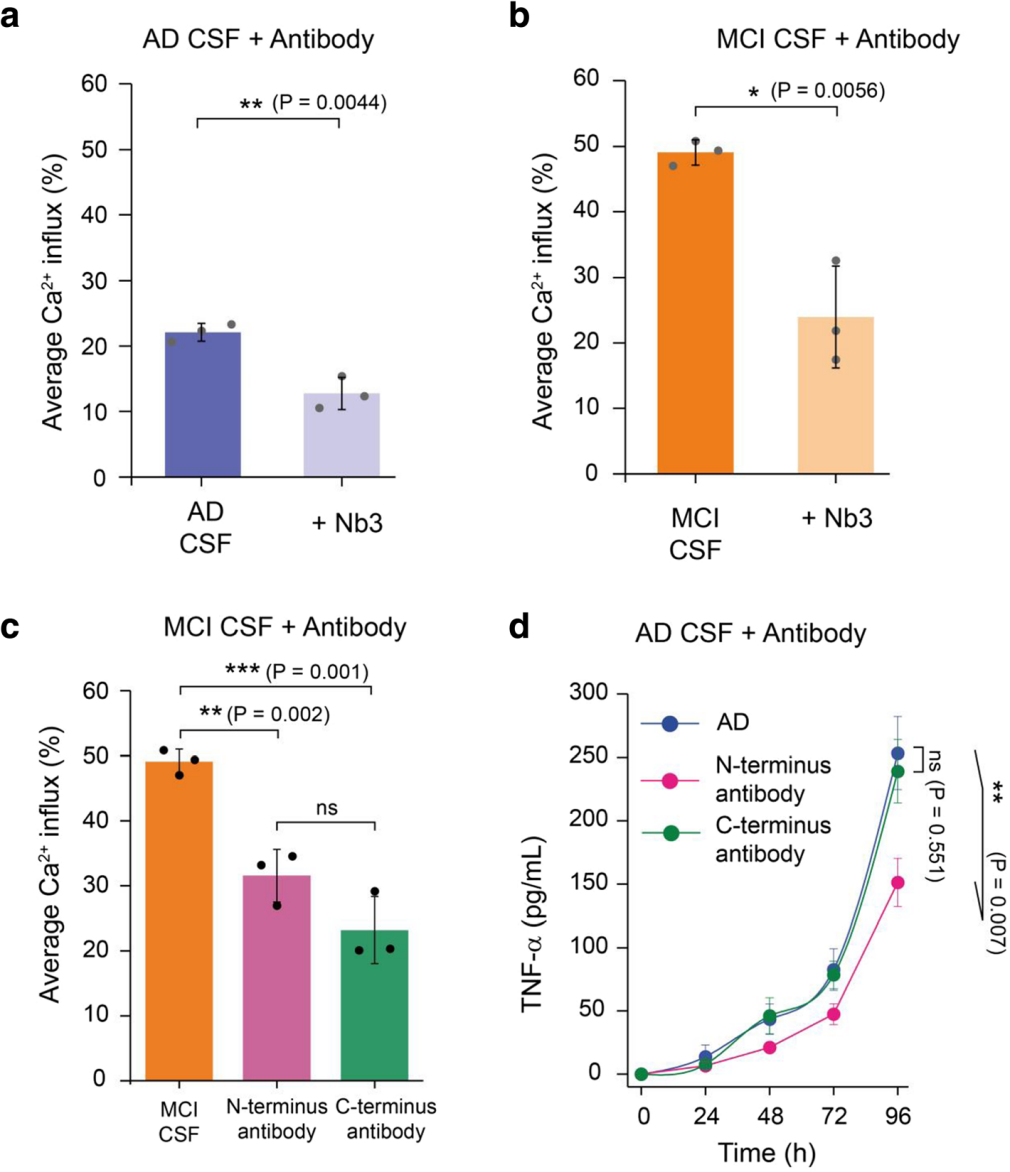
The toxic soluble aggregates present in MCI and AD CSF contain Aβ. A significant inhibition of the membrane permeabilisation of lipid membranes by (**a**) AD and (**b**) MCI CSF samples is caused by a nanobody Nb3 (300 nM) designed to bind to Aβ, indicating that some of the aggregates present in MCI and AD CSF contain Aβ. Nb3 recognises amino acids 17–28 of the Aβ sequence and shown to inhibit toxicity induced by protein aggregates of Aβ cerebrospinal fluid samples from AD patient (**c**) Both N-terminally and C-terminally targeting antibodies significantly inhibit MCI CSF-induced membrane permeabilisation. However, we do not find any significant difference between their activities**.** Error bars are the standard deviation among data points. Two sample unpaired t-tests were performed to compare the data sets (*n* = 3). **d** A N-terminal binding antibody (binding the region of residues 3–9 of Aβ42) is more potent at reducing the aggregate-induced inflammatory response than a C-terminal designed antibody (binding the region of residues 36–42 of Aβ42); *P* values are calculated using a two sample t-test to compare the inhibition by an N-terminally binding antibody and a C-terminally antibody at 96 h (n = 3). Error bars are the standard deviation among data points

**Fig. 3:**
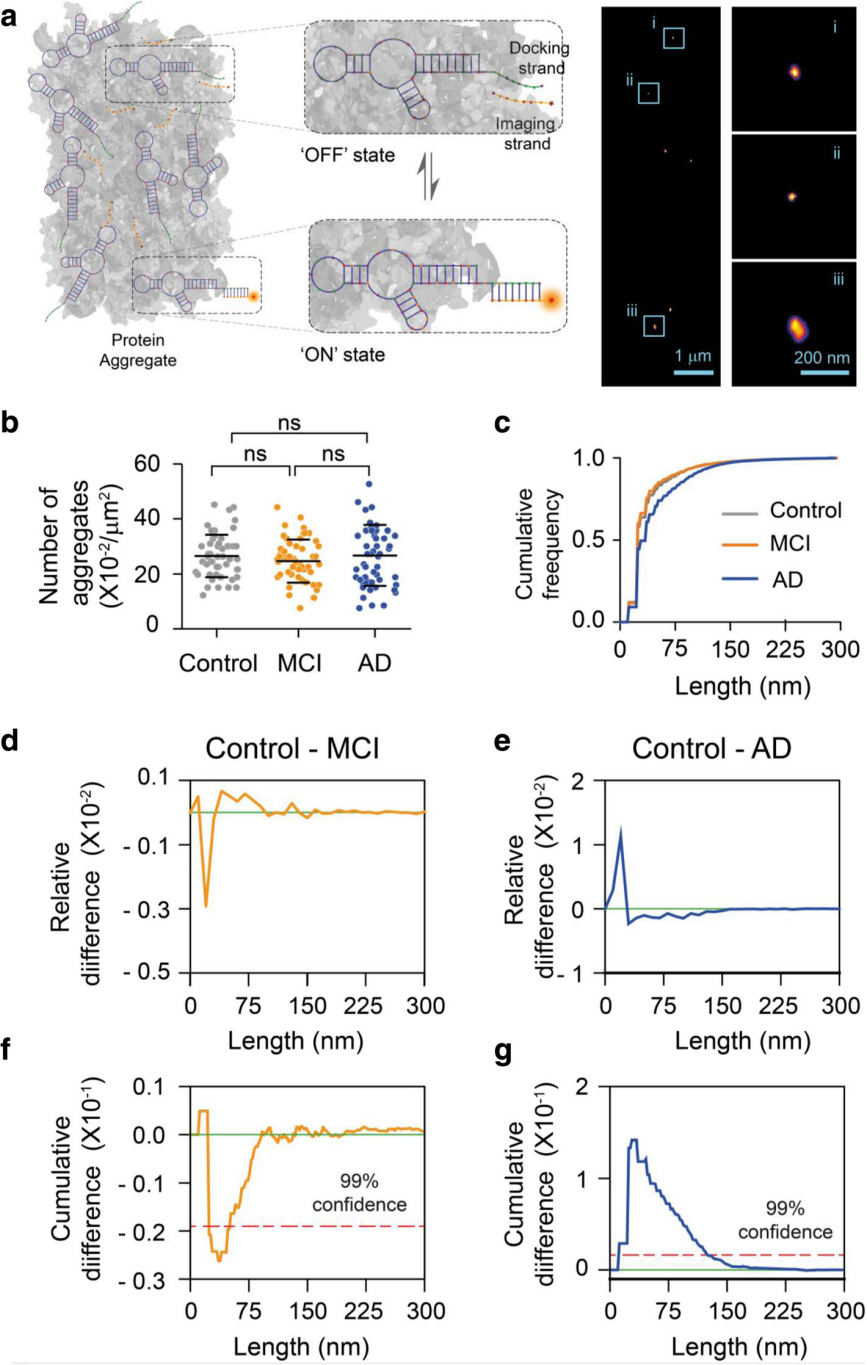
Super-resolution imaging of aggregates present in CSF using AD-PAINT. **a** Schematic of AD PAINT. Dye-labelled DNA imaging strands transiently bind to their complementary target sequence (docking strand), which is attached to a protein aggregate via aptamer. This transient binding between imaging and docking strand is detected. Repeated cycles of binding and unbinding allows a super-resolved image of individual protein aggregate present in CSF to be determined. The right image shows examples of super-resolved image of protein aggregates from CSF. Three individual protein aggregates present in CSF are enlarged. The lengths of the aggregates shown are: (i) 47 nm, (ii) 34 nm and (iii) 118 nm. **b** Number of aggregates present in control, MCI and AD CSF samples. Each point represents one field of view. **c** Cumulative frequency histograms of the size distributions of all aggregates measured. **d**, **e** Differences between normalised histograms of the size distributions of the indicated CSF samples. **f**, **g** Differences between the cumulative frequency histograms of the size distributions of the indicated CSF samples. The dotted line indicates 99% confidence using the Kolmogorov-Smirnov statistical test. (n = 6 AD, 6 MCI, 6 control CSF)

To assess the statistical significance of the difference among AD, MCI and control CSF for membrane permeability assay, we performed a two-sample t-test (unpaired) (Fig. 1b) using origin 9.0. We also used the same two-sample t-test (unpaired) assay to check if the antibodies significantly inhibit CSF-induced membrane permeabilisation (*n* = 3) (Fig. 2b. and Additional file 1: Figure S2). To determine the statistical significance of the results on the inflammatory response induced by AD, control and MCI CSF samples (Fig. 1d) and on the antibody-induced inhibition of the toxicity of AD CSF samples we also performed two-sample t-test (unpaired) at 96 h (n = 3) (Fig. 2a). The number of aggregates present in the CSF samples was tested using a one-way ANOVA with Tukey’s Multiple Comparison post-test (Fig. 3b and Additional file 1: Figure S1). Significance in the differences of the size distributions of the aggregates were evaluated using the Kolmogorov-Smirnov test (Fig. 3f, g).

## Results and discussions

### Soluble aggregates present in AD and MCI CSF induce toxicity by distinct mechanisms

We used CSF from individuals diagnosed with MCI, AD as well as healthy controls to perform a series of proof of concept experiments on a small set of clinical samples of CSF to explore if our assays could detect differences between the aggregates present in CSF at different stages of AD. We first set out to analyse the toxicity of the soluble aggregates in CSF by measuring their ability to permeabilise lipid membranes and induce an inflammatory response. As the non-specific binding of protein aggregates to lipid membranes is driven in particular by hydrophobic interactions, aggregates with more hydrophobic patches show increased toxicity via disruption and permeabilisation of the lipid membrane. To quantitatively measure how the soluble aggregates act in this manner, we used a recently developed biophysical assay, which has shown that physiological concentrations of soluble aggregates of Aβ can destabilise lipid membranes [9, 14]. For this assay, we immobilised thousands of single POPC vesicles (mean diameter - 200 nm) containing the Ca^2+^-specific dye, Cal-520, onto PEGylated glass cover slides via biotin-neutravidin linkage. When CSF samples are added, soluble aggregates present permeabilise the membrane of lipid vesicles and Ca^2+^ from the surrounding solution enter individual vesicles causing a change in the fluorescence intensity of the dye (Fig. 1a). The change in fluorescence intensity in these nano-sized vesicles is proportional to the number of ions that entered and can be quantified using TIRF microscopy [14, 20]. Using this sensitive method capable of detecting entry of a single Ca^2+^ ion, we found that aliquots of MCI CSF cause greater membrane permeabilisation compared to the AD and control CSF (Fig. 1b). By contrast, we found no significant difference in membrane permeation induced by AD and control CSF, in agreement with a previously reported study [11].

We instead observed the opposite trend in the CSF-induced pro-inflammatory response in glial cells (Fig. 1c). Physiological concentrations of protein aggregates can interact with specific membrane receptors in microglial cells and induce a proinflammatory response [19]. This response can be quantified by measuring secreted tumour necrosis factor alpha (TNF-ɑ), one of the pro-inflammatory cytokines that is produced, using an ELISA assay. To sensitively determine if CSF samples can induce a proinflammatory response, we added them to BV2 microglia cells and incubated for 5 days. These cells develop a sensitised response to the aggregates over time, leading to a detectable increase in TNF-α secretion, as we observed previously with synthetic aggregates of alpha synuclein [19]. Every 24 h, we took the supernatant above the cells and measured the TNF-α concentration. We found that over time the CSF aliquots can activate an innate immune response leading to the production of significant amounts of TNF-α. We observed that AD CSF samples induced a stronger inflammatory response than the MCI and control CSF samples (Fig. 1d). This inflammatory response induced by AD CSF was significantly blocked by *Rhodobacter sphaeroides* lipid A (RSLA), a known Toll-like receptor (TLR)-4 antagonist [33] and TAK-242, a small-molecule inhibitor that binds selectively and inhibits signalling of TLR-4 [23] showing that signalling is mediated by TLR-4 (Additional file 1: Figure S1).

### Soluble amyloid-β aggregates present in AD and MCI CSF are structurally distinct

We explored the feasibility of performing immunodepletions in CSF, using antibodies on beads, but found that the non-specific control antibody also reduced the number of detectable aggregates and the amount of membrane permeabilization to the same extent as the specific antibody. This was due to the non-specific binding of the low concentration of aggregates in the CSF to the hydrophobic beads. Therefore, to gain insight into the composition of the soluble aggregates responsible for the observed membrane permeabilisation we used several antibodies raised against Aβ to determine whether they could block the CSF-induced toxicity A relatively high dynamic range is required to perform these experiments so they were therefore performed on the three MCI and AD CSF samples that showed the highest level of membrane permeabilization. To this end, we employed a series of Aβ-specific antibodies which were previously shown to counteract the toxicity induced by soluble aggregates of Aβ both in vitro and in AD CSF. Firstly we used an antibody Nb3 which recognises the NAC region (aa17–24) of the Aβ sequence, and found it was able to significantly reduce the aggregate-induced membrane permeabilisation for both AD and MCI (Fig. 2a, b). It was used at a concentration of 300 nM, 20-fold higher than its K_d_ (~ 13 nM) [32]. We also used two different aggregate specific designed antibodies which were shown to target regions near the N- and C- termini (aa3–9 and 36–42, respectively) of the Aβ peptide to confirm our findings [25, 30]. We found that both the antibodies recognising the N-terminus and C-termini were both effective at reducing lipid membrane permeation (Fig. 2c), These results indicate that at least a portion of the toxic aggregates present in MCI and AD CSF are composed of Aβ. In contrast, a non-specific control antibody did not reduce membrane permeabilization Additional file 1: Figure S2). We also found that the antibody that targets the N-terminus of Aβ, but not the antibody that targets the C-terminus, efficiently inhibits an AD CSF-induced inflammatory response (Fig. 2d). The differential effects of N-terminal and C-terminal antibodies provide information not only on the composition but also on the structure of the soluble aggregates present in AD CSF. Mature fibrillar aggregates have a hydrophobic C-terminus which is inaccessible to C-terminal antibodies, while the N-terminus residues of aggregates are exposed [16, 28, 31]. These results suggest that the aggregates present in the CSF that are responsible for inducing membrane permeation are structurally distinct from those that induce an inflammatory response.

### Single aggregate super-resolution imaging shows the differing size distributions of soluble aggregates present in AD and MCI CSF

To examine the size and morphology of these soluble aggregates, we employed a newly developed super-resolution technique, aptamer DNA PAINT (ADPAINT) (Fig. 3a) [43]. The small size of the aptamer in combination with its remarkable specificity and affinity allows us to resolve aggregated structures of Aβ with a precision of approximately 20 nm. We have previously demonstrated the utility of this imaging technique by accurately identifying and nanoscopically characterising the shapes and sizes of the species formed during an aggregation reaction and in iPSC neurons [43]. Using this technique, we analysed the average number of aggregates present in the CSF samples and measured the size of individual aggregates (Fig. 3b,c), which ranged from 20 nm, limited by our resolution, to 300 nm. We did not find any significant difference in the total number of aggregated species present among MCI, AD and control CSF samples (6 each), a result that we confirmed using the amyloid-specific dye PFTAA (Additional file 1: Figure S3).

To examine the aggregate morphologies, we plotted the relative differences between normalised (Fig. 3d,e) and cumulative (Fig. 3f,g) histograms of the size distributions of aggregates present in control vs MCI and AD CSF samples. In the plots of relative differences negative values indicate that more aggregates of that size are present in the disease CSF, whereas positive values indicate more aggregates of that size are present in control CSF. Similarly, a negative slope indicates more aggregates of that size are present in disease CSF when looking at the cumulative differences. We found that, with 99% confidence, the number of small aggregates (< 50 nm) present in MCI CSF was higher than that found in control CSF (Fig. 3d,f). This result suggests that a relatively larger number of small aggregates might be accountable for enhanced membrane permeabilisation induced by MCI CSF. This finding agrees with previously reported studies that small sized Aβ aggregates are more hydrophobic and have greater tendencies to interact, permeabilise and cross the plasma membrane [6, 25]. In contrast, a ten-fold larger change was observed in the size distribution of AD CSF compared to control CSF, with a larger number of longer, mature aggregates (~ 40 to 200 nm) (Fig. 3e,g). Together, these results, in combination with the AD CSF-induced inflammation inhibition by an N-terminus antibody (Fig. 2a) suggest that relatively longer aggregates present in AD CSF might be responsible for the increased inflammatory response. Additionally, these data are also in agreement with previous reports on synthetic Aβ aggregates, which cannot undergo post-translational modifications. It was found that Aβ aggregates greater than 100 nm formed in artificial CSF can trigger an inflammatory response in microglial cells that can be blocked by an Aβ N-terminal region recognising antibody [8, 31, 42]. We also recently reported results that small aggregates of Aβ42 which form during the early stages of aggregation are more potent at membrane permeabilisation, whereas protofilament aggregates of similar length to the aggregates detected in CSF with height of 0.4–1 nm, which form at later stages of aggregation, are more effective at inducing inflammatory response in murine glial cells via TLR4 receptor [10]. Thus, the data on synthetic Aβ aggregates also supports the idea that longer Aβ aggregates in AD CSF cause neuroinflammation.

The increase in the relative proportion of large aggregates means that there is also a small increase in the total mass of Aβ present in AD CSF, as also previously detected by ELISAs. However, ELISA cannot measure the mass and aggregate number independently. This may explain why it is difficult to detect a clear difference between control and AD CSF since both factors contribute to the total aggregate mass detected in a given sample. This also suggests that the observed reduction in Aβ monomer concentration in AD CSF is partially due to this increased mass of aggregates, although this cannot account for the entire reduction and presumably there is also significant deposition of aggregates in the brain.

### Structural characterisation of soluble aggregates presents in CSF using high resolution atomic force microscopy (AFM) at the single aggregate level

**Fig. 4:**
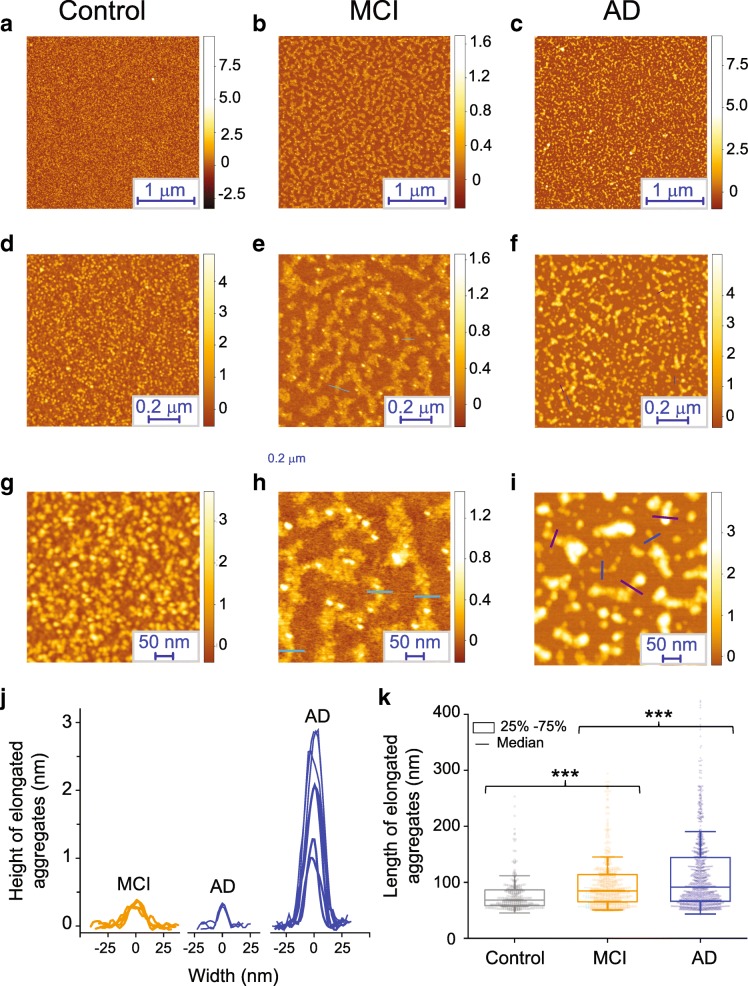
Characterization of the protein aggregates present in CSF samples at single aggregate level using AFM**. a**-**c** Representative AFM images of the aggregates present at different CSF samples with (**d**-**j**) a magnified example of the species present. **j**, **k** Statistical analysis of the cross-sectional length and heights of individual aggregates present in CSF samples; the length of the individual aggregates are shown as a box chart

To measure the heterogeneity and three-dimensional morphological properties of the protein aggregates present in the CSF samples, we utilised a phase controlled AFM technique to resolve the structures of individual protein aggregates at Angstrom resolution [35, 36] (Fig. 4). This allowed us to determine the heights of the individual species present in the CSF. For these experiments the samples of CSF were dried directly onto a mica surface. The control CSF sample showed the uniform presence of abundant spherical species, whereas both the MCI and AD samples showed the co-existence of spherical species and elongated aggregates (Fig. 4). We then performed a statistical analysis of their cross-sectional length and heights of individual aggregates [35]. The MCI CSF sample showed a relatively uniform population of elongated aggregates with a cross-sectional height ranging between 0.3–1 nm (Fig. 4b) and length 50–100 nm (Fig. 4c), larger than the typical length of species in the control sample (*p* < 0.001). The AD CSF samples showed the coexistence of highly heterogeneous elongated aggregates. These species belonged roughly to two populations of height, the first with cross sectional height ranging between 0.3–1 nm which is similar to the species present in MCI CSF aggregates, often referred to as protofilaments, and the second with cross-sectional height between 1 and 3 nm, often referred to as protofibrils. A larger cross-sectional aggregate height has been associated in vitro to an increased content of intermolecular β-sheet and maturity of amyloid structure [36]. The AD elongated aggregates are significantly longer than the MCI CSF sample (p < 0.001), ranging in length between 50 and 400 nm. This data is highly consistent with the super-resolution imaging data suggesting that the same species are being detected in both experiments. These data, in conjunction with the AD PAINT data, confirm that the protein aggregates present in AD CSF differ in structure from the aggregates present in MCI and control CSF. We cannot formally conclude that these species are Aβ aggregates since the AFM measurement is composition insensitive. However, we have shown that a higher proportion of longer aggregates are present in AD CSF and that this correlates with increased inflammation. Overall these findings suggest that long Aβ protofiibrils cause the increased inflammation detected in AD CSF. It has also been shown that that the protofibrillar aggregate of amyloid beta can cause the inflammation in murine BV2 cells [10]. Interestingly and in support of this conclusion, there is a crystal structure of TLR3, which is in the same family as TLR4, bound to an RNA dimer which is about 2 nm in diameter [29]. TLR3 signalling occurs when the RNA dimer is longer than 15 nm [26]. Since the long AD protofibrillar aggregates, have a cross-sectional height of round 2 nm, this provides a plausible explanation of how they cause TLR4 signalling in AD CSF.

## Conclusions

In conclusion, we have reported that there is a higher proportion of small aggregates that can cause increased membrane permeabilisation present in MCI CSF than in AD and control CSF, while in AD CSF there is an increased number of larger and longer aggregates, corresponding with enhanced neuroinflammation. These larger aggregates appear to be protofibrillar in nature, with distinct structural features from those of the smaller aggregates and appear to be the species that initiates cytokine production via TLR4 signalling.

It has to be noted that our study has been performed on a small number of samples and in future work it would be of interest to see if we observe the same changes in aggregate size distributions in bigger cohorts. If replicated then following the changes in aggregate size distribution provides a method to follow disease progression that does not require the measurement of the absolute number of aggregate species, with the proportion of larger aggregates increasing. Our results indicate that as the disease progresses there are changes in the aggregate size distribution in CSF but not in the total number of species. It has been reported that there is a three-fold increase in Aβ aggregates deposited in the brain during the development of AD [34]. Taken together this suggests that there is a change in both aggregate number and size distribution in the brain during the development of AD, but only the change in aggregate size distribution is detectable in the CSF sampled in a lumber puncture. This change in aggregate size distribution may be important in the disease mechanism since it corresponds with a change in aggregate structure and changes the dominant mechanism of aggregate-induced cellular toxicity. This in turn suggests that a combination of therapeutic agents targeting aggregated species with varying size and morphology, rather than a single agent targeting a single structure of one toxic form of Aβ may be needed to develop effective treatments for AD. Lastly, our data shows that sensitive biophysical methods are now capable of characterising the aggregates present in human CSF at new levels of detail, providing an opportunity to determine what changes in aggregate size and structure correlate with different stages of disease and new information about the species that initiate and drive the development of AD.

## Additional file

Additional file 1: **Table S1.** Characterisation of the CSF samples used in this study. **Figure S1** Toll like receptor 4 (TLR4) antagonists block AD CSF-induced aggregate induced inflammation. **Figure S2** a portion of the toxic aggregates present in MCI and AD CSF are composed of Aβ To understand the composition of toxic aggregate present in human CSF. we employed a series of Aβ-specific antibody which are known to counteract the toxicity induced by soluble aggregates of Aβ. **Figure S3** Detection of aggregates present in control, MCI and AD CSF using pFTAA. (DOCX 197 kb)

## Data Availability

All data and codes are available from the authors upon reasonable request.

## Abbreviation

AD: Alzheimer’s disease
AFM: Atomic force microscopy
CDR: Clinical dementia rating
CSF: Cerebrospinal fluid
ELISA: Enzyme-linked immunosorbent assay
MCI: Mild cognitive impairment
PET: Amyloid-β (Aβ) Positron-emission tomography
pFTAA: Pentameric formyl thiophene acetic acid
RSLA: Rhodobacter sphaeroides lipid A
TIRF: Total internal reflection fluorescence
TLR: Toll-like receptor
TNF-ɑ: Tumour necrosis factor alpha

## References

[1] Aprile Francesco A., Sormanni Pietro, Perni Michele, Arosio Paolo, Linse Sara, Knowles Tuomas P. J., Dobson Christopher M., Vendruscolo Michele (2017). Selective targeting of primary and secondary nucleation pathways in Aβ42 aggregation using a rational antibody scanning method. Science Advances.

[2] Benilova I, Karran E, De Strooper B (2012). The toxic Aβ oligomer and Alzheimer’s disease: an emperor in need of clothes. Nat Neurosci.

[3] Bjerke M, Kern S, Blennow K, Zetterberg H, Waern M, Börjesson-Hanson A (2016). Cerebrospinal Fluid Fatty Acid-Binding Protein 3 is Related to Dementia Development in a Population-Based Sample of Older Adult Women Followed for 8 Years. J Alzheimer’s Dis.

[4] Blennow K (2004). Cerebrospinal fluid protein biomarkers for Alzheimer’s disease. NeuroRX.

[5] Blennow K, Hampel H, Weiner M, Zetterberg H (2010). Cerebrospinal fluid and plasma biomarkers in Alzheimer disease. Nat rev Neurol.

[6] Campioni S, Mannini B, Zampagni M, Pensalfini A, Parrini C, Evangelisti E (2010). A causative link between the structure of aberrant protein oligomers and their toxicity. Nat Chem Biol.

[7] Chakrabarty Paramita, Li Andrew, Ladd Thomas B., Strickland Michael R., Koller Emily J., Burgess Jeremy D., Funk Cory C., Cruz Pedro E., Allen Mariet, Yaroshenko Mariya, Wang Xue, Younkin Curtis, Reddy Joseph, Lohrer Benjamin, Mehrke Leonie, Moore Brenda D., Liu Xuefei, Ceballos-Diaz Carolina, Rosario Awilda M., Medway Christopher, Janus Christopher, Li Hong-Dong, Dickson Dennis W., Giasson Benoit I., Price Nathan D., Younkin Steven G., Ertekin-Taner Nilüfer, Golde Todd E. (2018). TLR5 decoy receptor as a novel anti-amyloid therapeutic for Alzheimer’s disease. The Journal of Experimental Medicine.

[8] Colvin BA, Rogers VA, Kulas JA, Ridgway EA, Amtashar FS, Combs CK (2017). The conformational epitope for a new Aβ42 protofibril-selective antibody partially overlaps with the peptide N-terminal region. J Neurochem.

[9] De S, Klenerman D (2019) Imaging individual protein aggregates to follow aggregation and determine the role of aggregates in neurodegenerative disease. Biochim Biophys Acta Proteins Proteomics Available from: https://www.sciencedirect.com/science/article/pii/S1570963919300044 10.1016/j.bbapap.2018.12.010 PMC6676340 30611780

[10] De S, Wirthensohn DC, Flagmeier P, Hughes C, Aprile FA, Ruggeri FS (2019). Different soluble aggregates of Aβ42 can give rise to cellular toxicity through different mechanisms. Nat Commun.

[11] Drews A, De S, Flagmeier P, Wirthensohn D, Chen W-H, Whiten D (2017). Inhibiting the Ca2+ influx induced by human CSF. Cell Rep.

[12] Edelstein AD, Tsuchida MA, Amodaj N, Pinkard H, Vale RD, Stuurman N (2014). Advanced methods of microscope control using μManager software. J Biol Methods.

[13] Fidani Liana, Goulas Antonis, Mirtsou Vassiliki, Petersen Ronald C, Tangalos Eric, Crook Richard, Hardy John (2002). Interleukin-1A polymorphism is not associated with late onset Alzheimer's disease. Neuroscience Letters.

[14] Flagmeier P, De S, Wirthensohn DC, Lee SF, Vincke C, Muyldermans S (2017). Ultrasensitive Measurement of Ca2+ Influx into Lipid Vesicles Induced by Protein Aggregates. Angew Chemie - Int Ed.

[15] Fusco G, Chen SW, Williamson PTF, Cascella R, Perni M, Jarvis JA (2017). Structural basis of membrane disruption and cellular toxicity by α-synuclein oligomers. Science.

[16] Gremer Lothar, Schölzel Daniel, Schenk Carla, Reinartz Elke, Labahn Jörg, Ravelli Raimond B. G., Tusche Markus, Lopez-Iglesias Carmen, Hoyer Wolfgang, Heise Henrike, Willbold Dieter, Schröder Gunnar F. (2017). Fibril structure of amyloid-β(1–42) by cryo–electron microscopy. Science.

[17] Hansson Oskar, Seibyl John, Stomrud Erik, Zetterberg Henrik, Trojanowski John Q., Bittner Tobias, Lifke Valeria, Corradini Veronika, Eichenlaub Udo, Batrla Richard, Buck Katharina, Zink Katharina, Rabe Christina, Blennow Kaj, Shaw Leslie M. (2018). CSF biomarkers of Alzheimer's disease concord with amyloid-β PET and predict clinical progression: A study of fully automated immunoassays in BioFINDER and ADNI cohorts. Alzheimer's & Dementia.

[18] Heneka Michael T, Carson Monica J, Khoury Joseph El, Landreth Gary E, Brosseron Frederic, Feinstein Douglas L, Jacobs Andreas H, Wyss-Coray Tony, Vitorica Javier, Ransohoff Richard M, Herrup Karl, Frautschy Sally A, Finsen Bente, Brown Guy C, Verkhratsky Alexei, Yamanaka Koji, Koistinaho Jari, Latz Eicke, Halle Annett, Petzold Gabor C, Town Terrence, Morgan Dave, Shinohara Mari L, Perry V Hugh, Holmes Clive, Bazan Nicolas G, Brooks David J, Hunot Stéphane, Joseph Bertrand, Deigendesch Nikolaus, Garaschuk Olga, Boddeke Erik, Dinarello Charles A, Breitner John C, Cole Greg M, Golenbock Douglas T, Kummer Markus P (2015). Neuroinflammation in Alzheimer's disease. The Lancet Neurology.

[19] Hughes CD, Choi ML, Ryten M, Hopkins L, Drews A, Botía JA (2019). Picomolar concentrations of oligomeric alpha-synuclein sensitizes TLR4 to play an initiating role in Parkinson’s disease pathogenesis. Acta Neuropathol.

[20] Iljina M, Dear AJ, Garcia GA, De S, Tosatto L, Flagmeier P (2018). Quantifying co-oligomer formation by α-Synuclein. ACS Nano.

[21] Jack CR, Bennett DA, Blennow K, Carrillo MC, Dunn B, Haeberlein SB (2018). NIA-AA research framework: toward a biological definition of Alzheimer’s disease. Alzheimer’s Dement.

[22] Jekel K, Damian M, Wattmo C, Hausner L, Bullock R, Connelly PJ (2015). Mild cognitive impairment and deficits in instrumental activities of daily living: a systematic review. Alzheimers res Ther.

[23] Kawamoto T, Ii M, Kitazaki T, Iizawa Y, Kimura H (2008). TAK-242 selectively \resses Toll-like receptor 4-signaling mediated by the intracellular domain. Eur J Pharmacol.

[24] Kern S, Zetterberg H, Kern J, Zettergren A, Waern M, Höglund K (2018). Prevalence of preclinical Alzheimer disease. Neurology.

[25] Kremer JJ, Pallitto MM, Sklansky DJ, Murphy RM (2000). Correlation of β-amyloid aggregate size and hydrophobicity with decreased bilayer fluidity of model membranes. Biochemistry.

[26] Leonard JN, Ghirlando R, Askins J, Bell JK, Margulies DH, Davies DR (2008). The TLR3 signaling complex forms by cooperative receptor dimerization. Proc Natl Acad Sci.

[27] Lesné S, Koh MT, Kotilinek L, Kayed R, Glabe CG, Yang A (2006). A specific amyloid-beta protein assembly in the brain impairs memory. Nature.

[28] Li S, Jin M, Liu L, Dang Y, Ostaszewski BL, Selkoe DJ (2018). Decoding the synaptic dysfunction of bioactive human AD brain soluble Aβ to inspire novel therapeutic avenues for Alzheimer’s disease. Acta Neuropathol Commun.

[29] Liu L, Botos I, Wang Y, Leonard JN, Shiloach J, Segal DM (2008). Structural Basis of Toll-Like Receptor 3 Signaling with Double-Stranded RNA. Science.

[30] Mc Donald JM, Savva GM, Brayne C, Welzel AT, Forster G, Shankar GM (2010). The presence of sodium dodecyl sulphate-stable Aβ dimers is strongly associated with Alzheimer-type dementia. Brain.

[31] Paranjape GS, Terrill SE, Gouwens LK, Ruck BM, Nichols MR (2013). Amyloid-β (1–42) Protofibrils formed in modified artificial cerebrospinal fluid bind and activate microglia. J Neuroimmune Pharmacol.

[32] Paraschiv Gabriela, Vincke Cécile, Czaplewska Paulina, Manea Marilena, Muyldermans Serge, Przybylski Michael (2012). Epitope structure and binding affinity of single chain llama anti-β-amyloid antibodies revealed by proteolytic excision affinity-mass spectrometry. Journal of Molecular Recognition.

[33] Rallabhandi P, Phillips RL, Boukhvalova MS, Pletneva LM, Shirey KA, Gioannini TL (2012). Respiratory Syncytial Virus Fusion Protein-Induced Toll-Like Receptor 4 (TLR4) Signaling Is Inhibited by the TLR4 Antagonists Rhodobacter sphaeroides Lipopolysaccharide and Eritoran (E5564) and Requires Direct Interaction with MD-2. Pier G, editor. MBio.

[34] Roberts BR, Lind M, Wagen AZ, Rembach A, Frugier T, Li Q-X (2017). Biochemically-defined pools of amyloid-β in sporadic Alzheimer’s disease: correlation with amyloid PET. Brain.

[35] Ruggeri FS, Benedetti F, Knowles TPJ, Lashuel HA, Sekatskii S, Dietler G (2018). Identification and nanomechanical characterization of the fundamental single-strand protofilaments of amyloid α-synuclein fibrils. Proc Natl Acad Sci.

[36] Ruggeri FS, Longo G, Faggiano S, Lipiec E, Pastore A, Dietler G (2015). Infrared nanospectroscopy characterization of oligomeric and fibrillar aggregates during amyloid formation. Nat Commun.

[37] Rydberg Sterner Therese, Ahlner Felicia, Blennow Kaj, Dahlin-Ivanoff Synneve, Falk Hanna, Havstam Johansson Lena, Hoff Maria, Holm Mathias, Hörder Helena, Jacobsson Tina, Johansson Boo, Johansson Lena, Kern Jürgen, Kern Silke, Machado Alejandra, Mellqvist Fässberg Madeleine, Nilsson Johan, Ribbe Mats, Rothenberg Elisabet, Rydén Lina, Sadeghi André, Sacuiu Simona, Samuelsson Jessica, Sigström Robert, Skoog Johan, Thorvaldsson Valgeir, Waern Margda, Westman Eric, Wetterberg Hanna, Zetterberg Henrik, Zetterberg Madeleine, Zettergren Anna, Östling Svante, Skoog Ingmar (2018). The Gothenburg H70 Birth cohort study 2014–16: design, methods and study population. European Journal of Epidemiology.

[38] Savage MJ, Kalinina J, Wolfe A, Tugusheva K, Korn R, Cash-Mason T (2014). A sensitive aβ oligomer assay discriminates Alzheimer’s and aged control cerebrospinal fluid. J Neurosci.

[39] Scheltens Philip, Blennow Kaj, Breteler Monique M B, de Strooper Bart, Frisoni Giovanni B, Salloway Stephen, Van der Flier Wiesje Maria (2016). Alzheimer's disease. The Lancet.

[40] Shankar Ganesh M, Li Shaomin, Mehta Tapan H, Garcia-Munoz Amaya, Shepardson Nina E, Smith Imelda, Brett Francesca M, Farrell Michael A, Rowan Michael J, Lemere Cynthia A, Regan Ciaran M, Walsh Dominic M, Sabatini Bernardo L, Selkoe Dennis J (2008). Amyloid-β protein dimers isolated directly from Alzheimer's brains impair synaptic plasticity and memory. Nature Medicine.

[41] Taylor CG, Meisl G, Horrocks MH, Zetterberg H, Knowles TPJ, Klenerman D (2018). Extrinsic amyloid-binding dyes for detection of individual protein aggregates in solution. Anal Chem.

[42] Terrill-Usery SE, Colvin BA, Davenport RE, Nichols MR (2016). Aβ40 has a subtle effect on Aβ42 protofibril formation, but to a lesser degree than Aβ42 concentration, in Aβ42/Aβ40 mixtures. Arch Biochem Biophys.

[43] Whiten DR, Zuo Y, Calo L, Choi M-L, De S, Flagmeier P (2018). Nanoscopic characterisation of individual endogenous protein aggregates in human neuronal cells. ChemBioChem.

