# Supplementary material for: Soluble aggregates present in cerebrospinal fluid change in size and mechanism of toxicity during Alzheimer’s disease progression
Source: Acta Neuropathol Commun. 2019 Jul 26;7:120. doi: 10.1186/s40478-019-0777-4 (PMC6659275; doi:10.1186/s40478-019-0777-4)
Supplement: Supplementary file 1 — Table S1. Characterisation of the CSF samples used in this study. Figure S1 Toll like receptor 4 (TLR4) antagonists block AD CSF-induced aggregate induced inflammation. Figure S2 a portion of the toxic aggregates present in MCI and AD CSF are composed of Aβ To understand the composition of toxic aggregate present in human CSF. we employed a series of Aβ-specific antibody which are known to counteract the toxicity induced by soluble aggregates of Aβ. Figure S3 Detection of aggregates present in control, MCI and AD CSF using pFTAA. (DOCX 197 kb) [file 40478_2019_777_MOESM1_ESM.docx]

Supplementary Materials:

**Soluble aggregates present in cerebrospinal fluid change in size and mechanism of toxicity during Alzheimer’s disease progression**

Suman De^1,11^, Daniel R. Whiten ^1,11^, Francesco S. Ruggeri^1,2,11^, Craig Hughes^3,11^, Margarida Rodrigues^1^, Dimitrios I. Sideris^1^, Christopher G. Taylor^1^, Francesco A. Aprile^1,2^, Serge Muyldermans^4^, Tuomas P. J. Knowles^1,2^, Michele Vendruscolo^1,2^, Clare Bryant^3^, Kaj Blennow^5,6^, Ingmar Skoog^7^, Silke Kern^7^, Henrik Zetterberg^5,6,8,9^, David Klenerman^1,10^*

^1^Department of Chemistry, University of Cambridge, Cambridge CB2 1EW, United Kingdom

^2^Centre for Misfolding Diseases, University of Cambridge, Cambridge CB2 1EW, United Kingdom

^3^Department of Veterinary Medicine, University of Cambridge, Cambridge, CB3 0ES, United Kingdom

^4^Laboratory of Cellular and Molecular Immunology, Vrije Universiteit Brussel, Brussels, Belgium.

^5^Clinical Neurochemistry Laboratory, Unit of Department of Psychiatry and Neurochemistry, Institute of Neuroscience and Physiology, the Sahlgrenska Academy at the University of Gothenburg, Sweden

^6^Clinical Neurochemistry Laboratory, Sahlgrenska University Hospital, Mölndal, Sweden

^7^Neuropsychiatric Epidemiology Unit, Department of Psychiatry and Neurochemistry, Institute of Neuroscience and Physiology, the Sahlgrenska Academy at the University of Gothenburg, Sweden

^8^Department of Neurodegenerative Disease, UCL Queen Square Institute of Neurology, University College London, Queen Square, London, UK

^9^UK Dementia Research Institute at University College London, London, UK

^10^UK Dementia Research Institute at University of Cambridge, Cambridge CB2 0XY, United Kingdom

^11^These authors contributed equally

*Correspondence to D.K. ([dk10012@cam.ac.uk](mailto:dk10012@cam.ac.uk))

|  | Sample No | Total Tau ng/L | Aβ ng/L | P -tau ng/L | Age | Gender | CDR |
| --- | --- | --- | --- | --- | --- | --- | --- |
| **AD** | 1 | 1400 | 270 | 155 | 74 | F |  |
|  | 2 | 769 | 348 | 94 | 59 | F |  |
|  | 3 | 763 | 302 | 86 | 81 | M |  |
|  | 4 | 836 | 507 | 89 | 69 | F |  |
|  | 5 | 1090 | 492 | 109 | 69 | F |  |
|  | 6 | 713 | 518 | 84 | 79 | F |  |
|  | 7 | 1030 | 540 | 197 | 66 | M |  |
|  | 8 | 1290 | 447 | 203 | 70 | F |  |
|  | 9 | 597 | 581 | 85 | 73 | M |  |
|  | 10 | 1010 | 552 | 92 | 82 | F |  |
| **MCI** | 11 | 634 | 436 | 81 | 70 | F | 0.5 |
|  | 12 | 554 | 258 | 73 | 70 | F | 0.5 |
|  | 13 | 362 | 463 | 50 | 70 | M | 0.5 |
|  | 14 | 354 | 295 | 67 | 70 | M | 0.5 |
|  | 15 | 566 | 371 | 78 | 70 | M | 0.5 |
|  | 16 | 384 | 345 | 49 | 70 | F | 0.5 |
| **Control** | 17 | 231 | 728 | 36 | 70 | F | 0.0 |
|  | 18 | 265 | 838 | 38 | 70 | F | 0.0 |
|  | 19 | 205 | 724 | 36 | 70 | F | 0.0 |
|  | 20 | 265 | 762 | 44 | 70 | F | 0.0 |
|  | 21 | 286 | 1000 | 49 | 70 | M | 0.0 |
|  | 22 | 252 | 792 | 36 | 70 | M | 0.0 |

**Table S1. Characterisation of the CSF samples used in this study.**


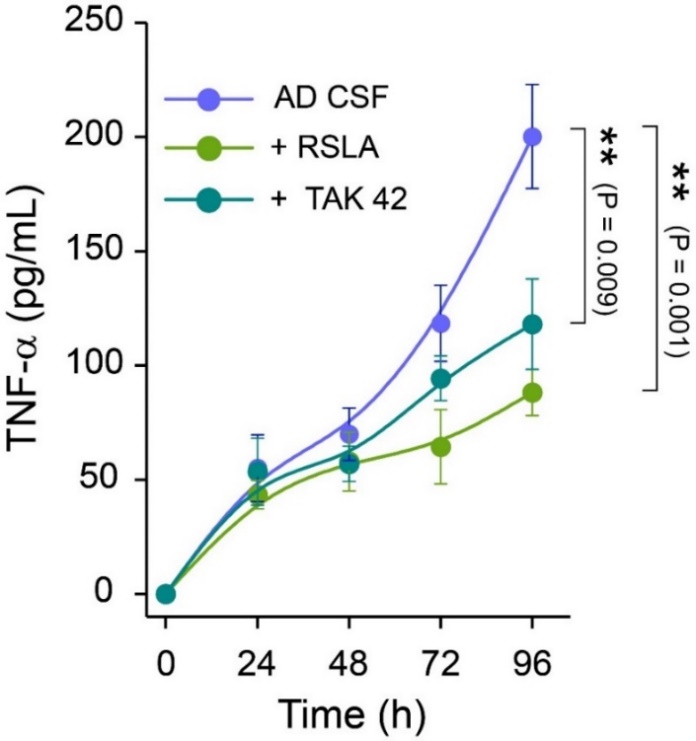


**Figure S1: Toll like receptor 4 (TLR4) antagonists block AD CSF-induced aggregate induced inflammation.** TAK-242, a small molecule inhibitor of TLR-4 and a known TLR4 antagonist *Rhodobacter sphaeroides* lipid A (RSLA) inhibits the AD CSF-induced inflammatory response by selectively binding to TLR4 and disrupting the interactions of TLR4 with its adaptor molecules (n=3, error bars is standard deviation). One way annova followed by post-hoc turkey were performed to compare the data sets.


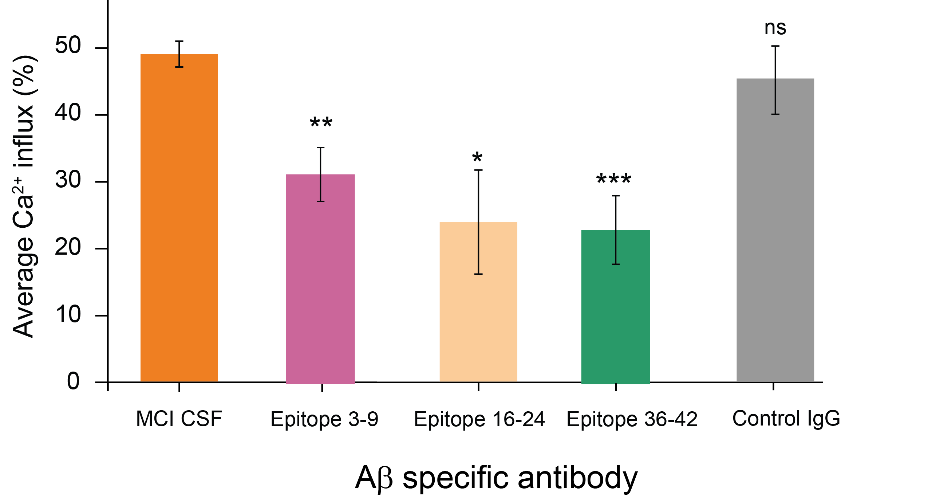


**Figure S2. a portion of the toxic aggregates present in MCI and AD CSF are composed of Aβ** To understand the composition of toxic aggregate present in human CSF. we employed a series of Aβ-specific antibody which are known to counteract the toxicity induced by soluble aggregates of Aβ
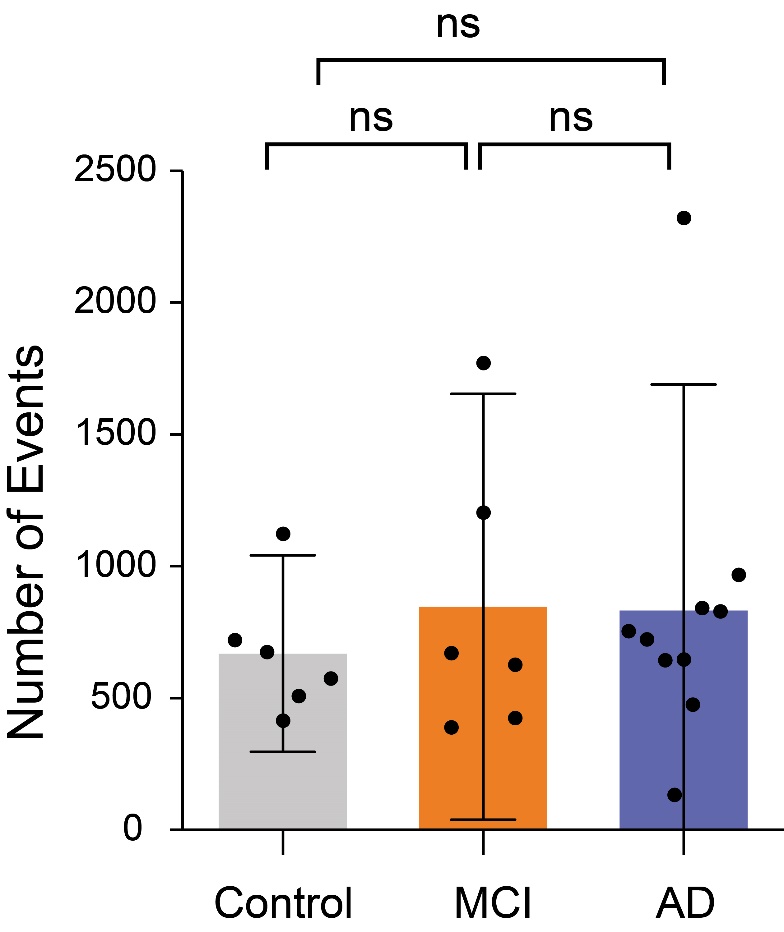


**Figure S3. Detection of aggregates present in control, MCI and AD CSF using pFTAA.** Pentameric formylthiophene acetic acid (pFTAA) is known to bind amyloid aggregates with high affinity. There is no significant difference in the number of pFTAA-active species in control, MCI and AD CSF. One way annova followed by post-hoc turkey were performed to compare the data sets
